# A rare and unusual new bittiine genus with two new species from the South Pacific (Cerithiidae, Gastropoda)

**DOI:** 10.3897/zookeys.758.25100

**Published:** 2018-05-14

**Authors:** Ellen E. Strong, Philippe Bouchet

**Affiliations:** 1 PO Box 37012, MRC 163, National Museum of Natural History, Smithsonian Institution, Washington, DC 20013-7012, USA; 2 Institut de Systématique, Évolution, Biodiversité, ISYEB, UMR7205 (CNRS, EPHE, MNHN, UPMC), Muséum National d’Histoire Naturelle, Sorbonne Universités, 43 Rue Cuvier, 75231 Paris Cedex 05, France

**Keywords:** Bittiinae, new genus, new species, marine, DNA

## Abstract

A new genus, *Limatium* **gen. n.**, and two new species, *L. pagodula* **sp. n.** and *L. aureum* **sp. n.** are described, found on outer slopes of barrier reefs and fringing reefs in the South Pacific. They are rare for cerithiids, which typically occur in large populations. The two new species are represented by 108 specimens sampled over a period of 30 years, only 16 of which were collected alive. Three subadults from the Philippines and Vanuatu likely represent a third species. In addition to their rarity, *Limatium* species are atypical for cerithiids in their smooth, polished, honey to golden brown shells with distinctive white fascioles extending suture to suture. The radula presents a unique morphology that does not readily suggest an affinity to any of the cerithiid subfamilies. Two live-collected specimens, one of each species and designated as holotypes, were preserved in 95% ethanol and sequenced. Bayesian analysis of partial COI and 16S rDNA sequences demonstrates a placement in the Bittiinae, further extending our morphological concept of the subfamily.

## Introduction

The Cerithiidae is one of 19 families of Cerithioidea currently accepted, and with 219 species considered valid, it is one of the most diverse (Bouchet et al. 2017, MolluscaBase 2018). Members are distributed worldwide from tropical to temperate biotopes, most frequently in shallow waters, with a few species that extend into bathyal depths. As far as is known, most species are microherbivorous grazers and usually occur in large populations (e.g., Houbrick 1992). The family is subdivided into three Recent subfamilies: Cerithiinae, Argyropezinae, and Bittiinae (Bouchet et al. 2017), with the Cerithiinae and Bittiinae containing the majority of the species. As discussed in Strong and Bouchet (2013, and references therein), there is presently no known diagnostic feature of shell morphology or internal anatomy that allows unambiguous placement in Cerithiinae versus Bittiinae. The only possible exception may be internal features of the midgut, but this requires further study to confirm (Strong and Bouchet 2013). The notion that bittiines may be distinguished by small adult size is a frequent misperception.

Unusual polished, golden shells, less than one centimeter in adult length, of an unfamiliar species first became known to us in material collected by the RAPA 2002 expedition to the Austral Islands. Since then, material comprising at least two species has been sorted from residues collected during campaigns led by the MNHN, with particularly rich sources of specimens from the MONTROUZIER expedition to New Caledonia in 1993, and the LIFOU 2000 expedition to the Loyalty Islands. The earliest specimens located thus far were collected during the MUSORSTOM 3 cruise to the Philippines in 1985, from the early years of the Tropical Deep Sea Benthos program (Bouchet et al. 2008). Despite readily identifiable as belonging to the Cerithiidae, their subfamily placement was unclear. Early collections were represented mainly by empty shells, with only a handful of live-collected specimens that had been subsequently dried. This hampered the use of anatomical dissections to explore their systematic placement; the unique radula did not suggest an affinity to any of the cerithiid subfamilies. The ability to assess their affinities more robustly was possible only with the collection of the first live-collected specimen preserved in ethanol during the Moorea Biocode Project in 2008. In 2013 a specimen of a second species was preserved for molecular analysis during the TUHAA PAE 2013 cruise to the Austral Islands. Based on this material, we here describe a new genus, *Limatium* n. gen., with two new species, *L. pagodula* sp. n. and *L. aureum* sp. n.

## Methods

The only two live-collected, fluid-preserved specimens were those used for sequencing and are the designated holotypes. The head-foot of each was removed through the aperture after drilling a small hole through the abapertural side of the penultimate whorl of the shell. The radulae of the holotypes were extracted and mounted for examination via scanning electron microscopy. However, the radula of *Limatium pagodula* sp. n. was teratological with thin, flimsy, poorly formed teeth along its length; no other live-collected material exists for this species and the operculum is unknown. In addition to the holotype of *Limatium aureum* sp. n., a second specimen live collected in Rapa and subsequently dried was used to extract an operculum and a radula for comparative purposes.

The three radulae and single operculum were tissue digested overnight in 100 µl of ATL lysis buffer (Qiagen, Inc.) containing ~50 µg of Proteinase-K, sonicated and rinsed in de-ionized water. Cleaned radulae were mounted directly on glass coverslips; the operculum was attached to the cover slip using a carbon adhesive tab. The cover slips were then attached to aluminum stubs with carbon adhesive tabs, coated with 25-30 nm gold/palladium (60/40), and photographed using an Apreo scanning electron microscope (FEI Company) at the NMNH. Specimens of subadults and juveniles were selected for examination of protoconchs. These shells were mounted on aluminum stubs with Elmer’s glue© and photographed uncoated in charge reduction mode using a Hitachi TM3000 scanning electron microscope (Hitachi High Technologies America, Inc.) also at the NMNH. Shells were photographed using a Canon EOS 50D camera with a Canon MP-E 65 mm f/2.8 1–5× macro lens and Canon MT-24EX macro twin light flash (Canon USA, Inc.).

The partial COI sequence for the holotype of *Limatium pagodula* sp. n., was produced under the Moorea Biocode Project (see Geller et al. 2013). For the other newly generated sequences, genomic DNA was extracted from roughly one cubic millimeter of 95% ethanol-preserved foot tissue using an automated phenol:chloroform extraction on the Autogenprep965 (Autogen, Holliston, MA) using the mouse tail tissue protocol with a final elution volume of 50 µl. A 658 base pair (bp) fragment of the cytochrome *c* oxidase I (COI) gene was amplified using degenerate Folmer primers (dgLCO/dgHCO) (Meyer et al. 2005) with M13 tails, and using JGLCO (Geller et al. 2013) in combination with C1-N-2191R (*aka* “Nancy”) (Simon et al. 1994). A ~510 bp fragment of the 16S rRNA gene was amplified using the universal primers 16SAR/BR (Palumbi et al. 1991). PCR reactions were performed with 1 μl of undiluted DNA template in 20 μl volumes. Reaction volumes for COI contained 10 µl of Promega Go-Taq® Hotstart Master Mix (1 unit Promega Go-Taq®, 400 μM dNTPs, 4 mM MgCl_2_), 0.3 µl 10 µM of each primer, 0.25 µg/μl of BSA, and 1.25% DMSO. Amplification consisted of an initial denaturation step at 95 °C for 7 min, followed by 45 cycles of denaturation at 95 °C for 45 s, annealing at 42 °C for 45 s, extension at 72 °C for 1 min and a final extension at 72 °C for 5 min. Reaction volumes for 16S, also in 20 μl volumes, contained 1 μl of undiluted template DNA, 1 unit Biolase DNA Polymerase (Bioline), 2 µl 10X reaction buffer, 500 μM dNTPs, 2 mM MgCl2, 0.25 μg/μl of BSA, and 0.3 µl 10 µM of each primer. Amplification consisted of an initial denaturation step at 95 °C for 5 min, followed by 35 cycles of denaturation at 95 °C for 30 s, annealing at 48 °C for 30 s, extension at 72 °C for 45 s and a final extension at 72 °C for 3 min. PCR products were purified prior to sequencing using the Exo-SAP-IT protocol (Amersham Biosciences, Piscataway, NY). Sequencing reactions were performed with 1 µl of purified PCR product, 1.75 µl BigDye buffer, and 0.5 µl BigDye (ABI, Foster City, CA), and run in the thermal cycler for 30 cycles of 30 s at 95 °C, 30 s at 50 °C, 4 min at 60 °C, and then held at 10 °C. Sequencing reactions were purified using Millipore Sephadex plates (MAHVN-4550, Millipore, Billerica, MA) per manufacturer’s instructions and analyzed on an ABI 3730XL DNA Analyzer Capillary Array.

Chromatograms were trimmed, assembled, and edited as necessary in Geneious 11.0.2. Sequences were aligned separately for each gene with ClustalX (Thompson et al. 1997) using default parameters as implemented in Geneious. COI was translated into amino acids to check for stop codons and frameshift mutations. All newly generated sequences have been deposited in GenBank (Table 1).

**Table 1.** Museum registration and GenBank accession numbers for specimens included in the phylogenetic analysis. Representatives of type species (or their subjective synonyms) of genera currently accepted as valid, indicated by ‘*’. Sequences previously published in the analysis of Strong and Bouchet (2013) in regular font, newly sequenced specimens in bold.

Species	Voucher	COI	16S	Locality
**Litiopidae**				
*Litiopa melanostoma* Rang, 1829 *	USNM 1199716	KC699870	KC699903	Marathon Key, Coco Plum Beach, Florida, USA, 24°43.45'N, 81°00.04'W.
**Cerithiidae**				
**Bittiinae**				
*Bittiolum varium* (Pfeiffer, 1840)	USNM 1199719	KC699852	KC699912	Sebastian Inlet, Florida, USA, 27°51.63'N, 80°26.95'W, 1 m.
*Bittium glareosum* Gould, 1861	MNHN IM-2009-29804	KC699853	KC699905	INHACA 2011, Stn. MS14, Baixo Danae, 25°54.5'S, 33°2.8'E, 23–26 m.
*Bittium impendens* (Hedley, 1899)	USNM 1199720	KC699854	KC699911	Shark’s Cove, Oahu, Hawaii, USA, 21°38.99'N, 158°3.8'W, 1 m.
*Bittium latreillei* (Payraudeau, 1826)	USNM 1199724	KC699855	KC699914	Espigon de Rocas, Benalmádena-Costa, Benalmádena, Spain 36°35.3'N, 04°31.7'W, 1–5 m.
***Bittium reticulatum* (da Costa, 1778)** *	**USNM 1462732**	**MH253703**	**MH253699**	**Espigon de Rocas, Benalmádena-Costa, Benalmádena, Spain 36°35.3'N, 04°31.7'W, 1–5 m..**
*Bittium simplex* (Jeffreys, 1867)	USNM 1199729	KC699856	KC699913	Strait of Gibraltar, Isla de Tarifa, Spain, 36°00.3'N, 05°36.53'W, intertidal.
*Cacozeliana granarium* (Kiener, 1842) *	USNM 1200194	KC699857	KC699904	Long Reef, Sydney, New South Wales, Australia, 33°44.6'S, 151°19.1'E, intertidal.
***Ittibittium houbricki* (Ponder, 1993)**	**MNHN IM-2013-42433**	**MH253702**	**MH253698**	**Cape Naturaliste, Eagle Bay, Western Australia**
*Ittibittium parcum* (Gould, 1861) *	USNM 1199730	KC699869	KC699902	Shark’s Cove, Oahu, Hawaii, USA, 21°39.16'N, 158°3.75'W, intertidal.
***Limatium aureum* sp. n. (holotype)**	**MNHN IM-2013-42460**	**MH253701**	**MH253697**	**TUHAA PAE 2013. Austral Islands, Maria. Pente externe récif barrière. 24 m. 21°47.8'S, 154°43'W.**
***Limatium pagodula* sp. n. (holotype)** *	**UF 427943**	**MH253700**		**Moorea Biocode Project. French Polynesia, Society Islands, Moorea. Haapiti, just NW of Matauvau Pass outer reef slope, brushed from under rubble. 20–22 m. -17.568°, -149.884°.**
*Pictorium koperbergi* (Schepman, 1907) *	MNHN IM-2009-26984	KC699871	KC699907	PANGLAO Marine Biodiversity Survey 2004, Stn. B10, Panglao I., Momo Beach, 9°36.5'N, 123°45.6'E, 3–14 m.
*Pictorium versicolor* Strong & Bouchet, 2013	MNHN IM-2009-26994	KC699874	KC699910	SANTO Marine Biodiversity Survey 2006, Stn. EP36, E Aoré I., Aimbuei Bay, 15°33.1'S-15°33.3'S, 167°12.4/12.7'E, 20–60 m.
*Pictorium violaceum* Strong & Bouchet, 2013	MNHN IM-2009-26986	KC699875	KC699909	SANTO Marine Biodiversity Survey 2006, Stn. EP36, E Aoré I., Aimbuei Bay, 15°33.1'S-15°33.3'S, 167°12.4/12.7'E, 20–60 m.
**Cerithiinae**				
*Cerithium atromarginatum* Dautzenberg & Bouge, 1933	USNM 1200200	KC699858	KC699899	Shark’s Cove, Oahu, Hawaii, USA, 21°39.16'N, 158°3.75'W, intertidal.
*Cerithium balteatum* Philippi, 1848	MNHN IM-2009-29697	KC699859	KC699889	SANTO Marine Biodiversity Survey 2006, Stn. DB53, Palikulo Bay, 15°28.8'S, 167°15.2'E, 5 m.
*Cerithium caeruleum* GB Sowerby II, 1855	MNHN IM-2009-27010	KC699860	KC699894	Atimo Vatae Madagascar “Deep South” Survey 2010, Stn. TM2, Cap Ranavalona, 25°4.3'S, 46°57.7'E, 0–1 m.
*Cerithium egenum* Gould, 1849	USNM 1200201	KC699861	KC699900	Shark’s Cove, Oahu, Hawaii, USA, 21°39.16'N, 158°3.75'W, intertidal.
*Cerithium lifuense* Melvill & Standen, 1895	MNHN IM-2009-29698	KC699862	KC699890	SANTO Marine Biodiversity Survey 2006, Stn. DB53, Palikulo Bay, 15°28.8'S, 167°15.2'E, 5 m.
*Cerithium munitum* GB Sowerby II, 1855	MNHN IM-2009-29699	KC699863	KC699891	SANTO Marine Biodiversity Survey 2006, Stn. DS4, Segond Channel, Coolidge wreck, 15°31.4'S, 167°14.1'E, 25 m.
*Cerithium nodulosum* Bruguière, 1792 *	MNHN IM-2009-29700	KC699864	KC699893	SANTO Marine Biodiversity Survey 2006, Stn. VM45, N Malo I., Andwélé rivulet, 15°37.7'S, 167°08.6'E, intertidal.
*Cerithium rostratum* A Adams, 1855	USNM 1200202	KC699865	KC699901	Shark’s Cove, Oahu, Hawaii, USA, 21°39.16'N, 158°3.75'W, intertidal.
*Cerithium salebrosum* GB Sowerby II, 1855	MNHN IM-2009-29701	KC699866	KC699892	SANTO Marine Biodiversity Survey 2006, Stn. DR64, Palikulo Bay, 15°27.6'S, 167°14.3'E, 6–35 m.
*Clypeomorus bifasciata* (GB Sowerby II, 1855) *	MNHN IM-2009-29702	KC699867	KC699888	SANTO Marine Biodiversity Survey 2006, Stn. ZM15, NW Malo, 15°38.1'S, 167°05.9'E, intertidal.
*Clypeomorus petrosa* (Wood, 1828)	MNHN IM-2009-29703	KC699868	KC699887	SANTO Marine Biodiversity Survey 2006, Stn. LM23, Segond Channel, vicinity of Maritime College, 15°31.5'S, 167°09.6'E, intertidal.
*Pseudovertagus aluco* (Linnaeus, 1758) *	MNHN IM-2009-29704	KC699883	KC699895	SANTO Marine Biodiversity Survey 2006, Stn. VM16, Bruat Channel, N coast of Malo I., 15°37.7'S, 167°11.0'E, intertidal.
*Rhinoclavis aspera* (Linnaeus, 1758)	MNHN IM-2009-29705	KC699884	KC699896	SANTO Marine Biodiversity Survey 2006, Stn. FR29, Palikulo Bay, 15°27.9'S, 167°14.6'E, 5–35 m.
*Rhinoclavis fasciata* (Bruguière, 1792)	MNHN IM-2009-29706	KC699885	KC699897	SANTO Marine Biodiversity Survey 2006, Stn. VM32, W Aésé I., 15°26.6'S, 167°15.2'E, intertidal.
*Rhinoclavis vertagus* (Linnaeus, 1767) *	MNHN IM-2009-29707	KC699886	KC699898	SANTO Marine Biodiversity Survey 2006, Stn. VM40, Surunda Bay, 15°27.7'S, 167°13.2'E, intertidal.

The best fit partitions and models for phylogenetic analyses were determined with PartitionFinder 1.1.1 (Lanfear et al. 2012) which favored the following scheme: COI: SYM+I+G, F81+I, HKY+G, for the first, second and third codon position, respectively; and GTR+I+G for 16S. A subset of the cerithiid dataset of Strong and Bouchet (2013) was used, augmented with newly generated COI and 16S sequences for *Ittibittium houbricki* (Ponder, 1993) and *Bittium reticulatum* (da Costa, 1778) (see Table 1). In addition to *Limatium*, the Bittiinae was represented by five of the 12 Recent genera currently accepted, four by their type species or their subjective synonyms (see Table 1). Bayesian analysis of the concatenated dataset for 29 cerithiids and one outgroup (Litiopidae) was inferred with MrBayes 3.2.6 (Ronquist and Huelsenbeck 2003) on the CIPRES Science Gateway, using the schemes and models indicated by PartitionFinder. Bayesian analyses, consisting of two independent replicates with four heated chains each (0.02), and three swaps per swapping cycle, were run for 50,000,000 Markov chain Monte Carlo (MCMC) generations with a sampling frequency of one tree every 1000 generations. The first 25% were discarded as burn-in. Tracer 1.6 (Rambaut et al. 2014) was used to assess MCMC convergence and to ensure that all ESS values exceeded 200. A majority rule consensus tree was constructed with the sumt command. Nodal support was assessed with posterior probability of each node.

## Results

### Systematics

#### Family CERITHIIDAE J. Fleming, 1822

##### Subfamily BITTIINAE Cossmann, 1906

###### Limatium gen. n.

Taxon classificationAnimaliaSorbeoconchaCerithiidae

http://zoobank.org/9FD47996-C44C-4D00-82A6-57FC9C619215

####### Type species.

*Limatium pagodula* sp. n.

####### Diagnosis.

Shells of small size, 6 to 7 mm in adult length on average, with smooth, polished surface, golden honey to dark brown in color. Rachidian with hexagonal to septagonal basal plate, squarish to rectangular, with elevated central portion with rounded, U-shaped lower margin; cutting edge with three, sharply pointed cusps. Operculum paucispiral with large, subcentral nucleus.

####### Etymology

From the Latin adjective *limatus, -a, -um*, meaning polished, and the ending -*ium* of many cerithiid genera. Gender neuter.

####### Ecology.

**Figure 1.**
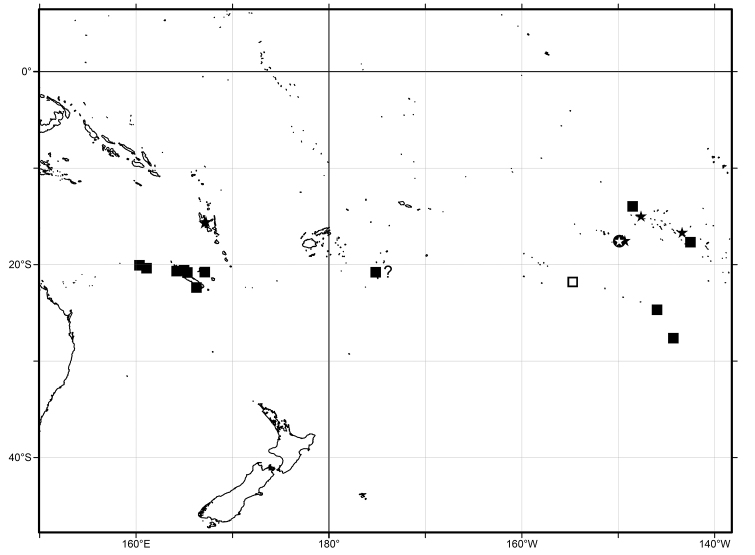
Distribution map of *Limatium pagodula* sp. n. («) and *Limatium aureum* sp. n. (■) in the South Pacific. Symbols with white fill indicate the type localities of the two species, in the Society (*L. pagodula*) and the Austral islands (*L. aureum*). The provisional record “■?” is for the unusually tall and narrow specimen from Tonga that was tentatively allocated to *L. aureum*.

All known specimens of *Limatium* come from the outer slope of barrier reefs or, in islands without a coral reef lagoon, from the slope of the fringing reefs in the South Pacific (Fig. 1). Not a single specimen has been collected within a coral reef lagoon. The few live-taken specimens come from a confirmed bathymetric range of 10-100 m, with empty shells, potentially carried downslope, occasionally dredged from deeper water.

####### Remarks.

*Limatium* differs from all other bittiine genera in the smooth, shiny, polished surface of the shell and its rich, golden honey to dark brown color. The two species known thus far are further distinguished by the distinctive white fascioles extending suture to suture and which may be a diagnostic feature of the genus, but further comparative material is required. No other bittiine is known to possess a rachidian basal plate that is hexagonal to septagonal in shape, with an elevated central portion; the cutting edge uniquely bears only three, sharply pointed, dagger-like cusps. The paucispiral operculum is also unique among bittiines as understood thus far.

###### Limatium pagodula sp. n.

Taxon classificationAnimaliaSorbeoconchaCerithiidae

http://zoobank.org/127FB8BA-1F31-4D61-A73B-5D05E8AC659B

Fig. 2

####### Type material.

**Figure 2.**
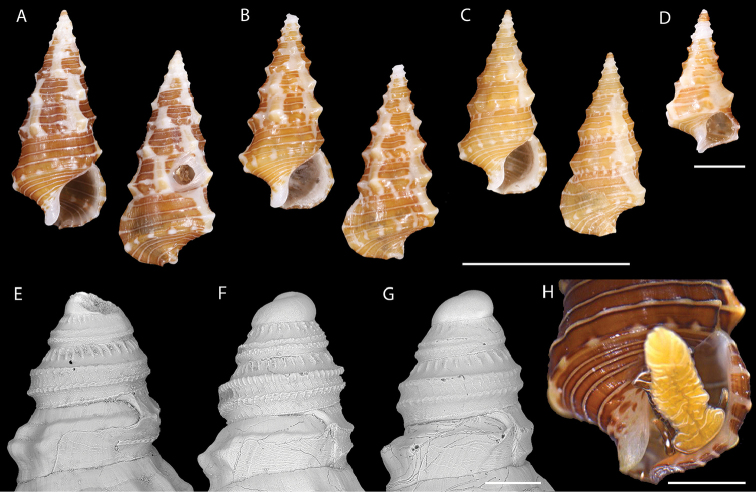
Shell and external morphology of *Limatium pagodula* sp. n. **A** Holotype, UF 427943. French Polynesia, Society Islands, Moorea **B** Paratype, USNM 1462731. Tuamotu Islands, Makemo, Pohue (ex. coll. Letourneux) **C** Paratype, MNHN IM-2014-6933. Vanuatu, Santo, W. of Tutuba I., SANTO 2006 stn. DS104 **D** Subadult. Tuamotu Islands, Makemo, Arikitamiro (coll. Letourneux) **E** Protoconch, USNM 1462729. Tahiti, grotte du chenal d’Arue (ex. coll. Letourneux) **F** Protoconch. Tuamotu Islands, Makemo, Arikitamiro (coll. Letourneux) (same as in 2D) **G** Protoconch, Tuamotu Islands, Makemo, Arikitamiro (coll. Letourneux) **H** Living animal, holotype UF 427943. French Polynesia, Society Islands, Moorea. Scale bars: 5 mm (**A–C**); 1 mm (**D, H**); 100 µm (**E–G**).

Holotype UF 427943 (Biocode No. MBIO19550, Specimen No. BMOO-03501,) (Fig. 2A, H); paratypes as listed below.

####### Type locality.

French Polynesia, Society Islands, Moorea. Haapiti, just NW of Matauvau Pass outer reef slope, brushed from under rubble, 17°34.1'S, 149°53.0'W, 20–22 m (Moorea Biocode; collector’s event ID MIB_087; *leg.* Chris Meyer & Christian McKeon; 20 October 2008).

####### Other material examined.

**FRENCH POLYNESIA**. SOCIETY IS: Tahiti, grotte du chenal d’Arue, ca. 17°31'S, 149°31.3'W, 12 m, 1 empty shell (dd), USNM 1462729 ex coll. Letourneux (Fig. 2E); Tahiti, faille d’Arue, 33 m, 1 dd, USNM 1462730 ex coll. Letourneux. TUAMOTU IS: Makemo, secteur de Pohue, 16°40.1'S, 143°22.5'W, 63 m, 1 dd, paratype USNM 1462731 ex coll. Letourneux (Fig. 2B); Makemo, passe Arikitamiro, ca. 16°37.1'S, 143°33.9'W, 45–54 m, 9 dd in coll. Letourneux (Fig. 2D, F; 6 not seen); Rangiroa, passe de Tiputa, ca. 14°58.0'S, 147°37.5'W, 100 m, 2 dd in coll. Letourneux (not seen). **VANUATU.** SANTO I.: SANTO 2006: stn. ZB9, W. Malo I., 15°40.6'S, 167°05.1'E, 5–7 m, 1 dd, MNHN uncatalogued. – Stn. DS104, W. of Tutuba I., Vunatavoa Bay, 15°34.1'S, 167°16'E, 10–80 m, 1 dd, paratype MNHN IM-2014-6933 (Fig. 2C).

####### Etymology.

From the Latin *pagoda*, with reference to the strongly angular whorls reminiscent of the upward curving roofs of Asian temples; *pagodula* is a diminutive, used as a noun in apposition.

####### Diagnosis.

**Shell [holotype, unless otherwise noted].** Shell narrow, slender, with high, conical spire, body whorl occupying ~45% of shell height, consisting of 9+ [first three whorls very encrusted] moderately convex but angular teleoconch whorls, suture impressed (Fig. 2A). Protoconch [very worn on holotype; description based on specimens illustrated in Fig. 2E–G] multispiral, of 2.5 whorls, with a sculpture of two strong, pustulose spiral keels at periphery, and a third, much lighter cord on the base, essentially covered by next whorl; strong, prosocline, axial riblets on sutural ramp, fading out abapically towards spiral keel; and irregular, elongated granules that cover the abapical part of the whorl and extend in between the two peripheral keels; protoconch/teleoconch transition sharp, with a lamellar terminal varix and a deeply indented sinusigera notch (Fig. 2E–G). Sculpture of teleoconch consisting of broad, poorly defined axial ribs forming a strong angular projection at adapical two-thirds of the whorl, crossed over by three (on body whorl 4) narrow, sharply defined spiral cords on exposed part of adult whorls, and a 4^th^, strongly raised, basal cord that is mostly covered by next whorl. Shell base slightly concave, with 7 unevenly spaced spiral cords. Siphonal canal very short, broadly open, constricted. Aperture circular-ovate, ~30% of shell height (in paratype, flaring and subquadrate, forming an angle where basal cord meets the outer lip). Anal canal indistinct. Columella concave with thinly callused columellar lip. Outer lip of aperture forming a terminal varix, subvertical on periphery, regularly convex on base. Shell surface smooth and shiny, as if waxed, color overall deep brown with broad, opaque white fascioles extending from suture to suture at irregular intervals, spiral cords on spire and base white, cord encircling the base brown with white blotches at regular intervals; columellar side of canal and columellar callosity white, parietal callosity transparent. Tip of teleoconch (first 1.5 whorl) white, protoconch dark brown. Dimensions: 6.50 × 2.65 mm. Average 5.98 ± 0.48 mm (n = 3).

**External anatomy.** Head-foot dark golden brown in color; cephalic tentacles with irregular white blotches and golden tips. Foot sole golden, with thin, transverse white lines, discontinuous across prominent longitudinal groove at midline; condition of pedal glands unknown. Epipodial skirt also with thin, transverse white to golden lines, present from propodium to large, projecting opercular lobe. Epipodial tentacles lacking.

**Radula.** The radula of the sequenced specimen was teratological, and we do not provide a detailed description or illustration. The gross features that were visible conform to those in *L. aureum* sp. n. (see below): a rachidian with broad hexagonal basal plate and elevated central portion with rounded base, cutting edge with three pointed cusps, lateral teeth with short lateral extensions roughly 1.5 times length of cutting edge, face of lateral teeth with buttress terminating in prominent, rounded knob midway down face of lateral teeth, outer edges of outer marginal teeth acuspate.

####### Distribution and ecology.

Society Islands and Tuamotus (French Polynesia); Vanuatu. Known only from the material examined. Only one specimen was collected alive, from the outer reef slope, brushed from under rubble, 20–22 m.

####### Remarks.

The sequenced specimen from Moorea is designated as holotype, although its outer lip is not fully mature. In the fully adult paratype from Santo (Fig. 2C), the axial white fascioles are fewer, but are aligned from one whorl to the next, and there is a strong varix one-half whorl before the aperture.

###### Limatium aureum sp. n.

Taxon classificationAnimaliaSorbeoconchaCerithiidae

http://zoobank.org/6EF8F349-E899-4E59-A93D-1CECB0CA47C0

Fig. 3A–H, K, L, N

####### Type material.

**Figure 3.**
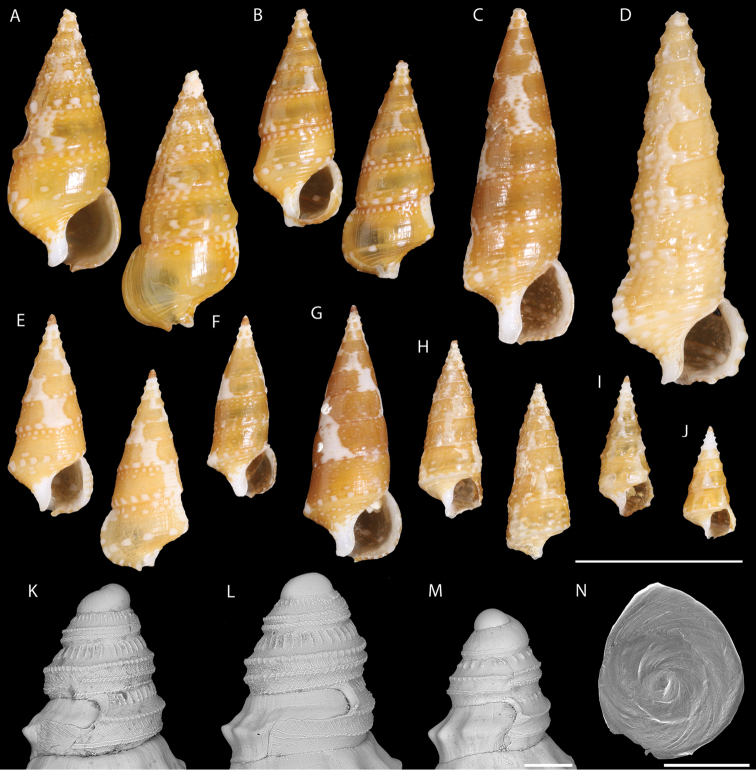
Shell and operculum morphology of *Limatium aureum* sp. n. (**A–H, K, L, N**) and *Limatium* sp. (**I, J, M**). *Limatium aureum* sp. n.: **A** Holotype, MNHN IM-2013-42460. Austral Islands, Maria **B** Austral Islands, Pointe Kauira, RAPA 2002 stn. 36 (MNHN IM-2014-6920) **C** New Caledonia, CORAIL 2 stn. DW26 (MNHN IM-2014-6921) **D** Tonga, between Eua and Tongatapu, BORDAU 2, stn. DW1512 (MNHN IM-2014-6922) **E** Austral Islands, NW of Tauna Islet, RAPA 2002 stn. 44 (MNHN IM-2014-6923) **F** New Caledonia, Grand Récif Aboré off Nouméa, MONTROUZIER stn. 1352 (MNHN IM-2014-6924). **G** Loyalty Islands, LIFOU 2000 stn. 1423 (MNHN IM-2014-6925) **H** Loyalty Islands, LIFOU 2000 stn. 1418 (MNHN IM-2014-6926) **K** Protoconch, New Caledonia, Grand Récif Aboré off Nouméa, MONTROUZIER stn. 1352 (MNHN IM-2014-6927) **L** Protoconch, Austral Islands, SE of Tauna Islet, RAPA 2002 stn. 8 (MNHN IM-2014-6928) **N** Operculum, Austral Islands, Pointe Kauira, RAPA 2002 stn. 36 (MNHN IM-2014-6829) (same as in 3B). *Limatium* sp.: **I** Philippines, W of Mindoro, MUSORSTOM 3 stn. DR117 (MNHN IM-2014-6830) **J** Vanuatu, Santo, W of Tutuba, SANTO 2006 stn. DS103 (MNHN IM-2014-6831) **M** Protoconch, Vanuatu, Santo, West of Tutuba I., SANTO 2006 stn. DS103 (MNHN IM-2014-6832) (same as in 3J). Scale bars: 5 mm (**A–J**); 100 µm (**K–M**); 500 µm (**N**).

Holotype MNHN IM-2013-42460 (Fig. 3A); paratypes as listed below.

####### Type locality.

Austral Islands, Maria I., outer slope of barrier reef, 21°47.8'S, 154°43'W, 24 m [TUHAA PAE 2013 cruise, stn. AMA02, field number PB16_BC855; *leg.* A. Fedosov; 5 April 2013].

####### Other material examined

(all in MNHN uncatalogued, except where noted): **FRENCH POLYNESIA**. AUSTRAL IS: RAPA Island expedition 2002: Stn. 6, off Baie de Ahurei, 27°36.8'S, 144°16.7'W, 42 m, 1 dd. – Stn. 8, SE of Tauna islet, 27°36.5'S, 144°17.7'W, 52–57 m, 7 dd (MNHN IM-2014-6928, Fig. 3L). – Stn. 28, Pointe Taekateke, 27°38.4'S, 144°20.6'W, 30 m, 1 dd. – Stn. 36, Pointe Kauira, 27°33.5'S, 144°20.8'W, 27 m, 1 live collected (lv), 2 dd (MNHN IM-2014-6920, IM-2014-6929, Fig. 3B, N). – Stn. 44, NW of Tauna islet, 27°36.3'S, 144°18.2'W, 30 m, 2 dd (MNHN IM-2014-6923, Fig. 3E). – BENTHAUS stn. DW1934, Banc Président Thiers, 24°40.6'S, 145°57.4'W, 560-1150 m, 1 dd. TUAMOTU IS: Makemo, secteur Pohue, 16°40.1'S, 143°22.5'W, 63 m, 1 dd, paratype USNM 1462727 ex coll. Letourneux; Makemo, passe Arikitamiro, ca. 16°37.1'S, 143°33.9'W, 45 m, 47 m and 54 m, 3 dd, in coll. Letourneux; Rangiroa, Passe de Tiputa, ca. 14°58.0'S, 147°37.5'W, 81 m and 100 m, 2 dd, USNM 1462728 ex coll. Letourneux. **NEW CALEDONIA.** Coral Sea. Lansdowne-Fairway Reefs. CORAIL 2 stn. DW26, 20°22'S, 161°05'E, 62 m, 1 lv (MNHN IM-2014-6921, Fig. 3C). – EBISCO stn. DW2622, 20°04'S, 160°21'E, 291–323 m, 1 dd. Mainland New Caledonia. LAGON Stn. 830, off Poindimié, 20°49'S, 165°19'E, 105–110 m, 4 dd. MONTROUZIER Stn. 1269, Récif Doiman off Touho, outer slope, 20°35.1'S, 165°08.1'E, 15–20 m, 4 dd. – Stn. 1331, Grand Récif de Koumac, outer slope, 20°40'-20°40.6'S, 164°11.2'-164°12.1'E, 55-57 m, 4 dd. – Stn. 1352, Grand Récif Aboré off Nouméa, outer slope, 22°22.2'S, 166°16.0/166°16.1'E, 27–35 m, 5 lv, 4 dd (MNHN IM-2014-6924, IM-2014-6927, Fig. 3F, K); – Stn. 1354, Grand Récif Aboré, outer slope, 22°22.3'S, 166°15.9'E, 27–37 m, 2 lv, 2 dd. BATHUS 1 stn. DW692, 20°35'S, 164°59'E, 140–150 m, 2 dd. **LOYALTY ISLANDS**: LIFOU 2000 Expedition, Baie du Santal: stn. 1418, 20°46.9'S, 167°07.9'E, 1–5 m, 1 dd (MNHN IM-2014-6926, Fig. 3H). – Stn. 1423, 20°54'S, 167°07.3'E, 12 m, 2 dd (MNHN IM-2014-6925, Fig. 3G). – Stn. 1429, 20°47.5'S, 167°07.1'E, 8–18 m, 2 dd. – Stn. 1432, 20°53.5'S, 167°02.7'E, 12–32 m, 2 dd. – Stn. 1434, 20°52.5'S, 167°08.1'E, 5–20 m, 2 dd. – Stn. 1441, 20°46.4'S, 167°02'E, 20 m, 2 dd. – Stn. 1442, 20°46.4'S, 167°02'E, 47 m, 1 dd. – Stn. 1443, 20°53.8'S, 167°07.3'E, 48–52 m, 3 dd. – Stn. 1445, 20°50.8'S, 167°09.7'E, 10–12 m, 1 dd. – Stn. 1448, 20°45.8'S, 167°01.6'E, 20 m, 4 dd. – Stn. 1449, 20°45.8'S, 167°01.6'E, 17 m, 1 dd. – Stn. 1450, 20°45.8'S, 167°01.6'E, 27–31 m, 1 dd. – Stn. 1451, 20°47.3'S, 167°06.8'E, 10–21 m, 3 dd. – Stn. 1453, 20°54.6'S, 167°02.1'E, 21–30 m, 1 dd. – Stn. 1454, 20°56.6'S, 167°02'E, 15–18 m, 2 dd. – Stn. 1455, 20°56.8'S, 167°02.7'E, 15–20 m, 1 lv, 1 dd. – Stn. 1456, 20°49.3'S, 167°10.4'E, 25–30 m, 1 lv, 1 dd. – Stn. 1457, 20°46.8'S, 167°02.8'E, 5–10 m, 2 lv, 1 dd. – Stn. 1461, 20°54'S, 167°02'E, 100–120 m, 1 lv, 1 dd. – Stn. 1469, 20°54.2'S, 167°00.4'E, 70–130 m, 2 dd.

####### Etymology.

Latin adjective *aureus*, -*a*, -*um*, meaning golden, with reference to the background color of the shell.

####### Diagnosis.

**Shell [holotype, unless otherwise noted].** Shell short, squat, with regular, conical spire, body whorl occupying ~46% of shell height, consisting of 9+ [tip of teleoconch and protoconch missing] rather flat teleoconch whorls, suture impressed (Fig. 3A). Protoconch [description based on specimens illustrated in Fig. 3K, L] multispiral, of 2.5 whorls, with a complex sculpture of two strongly pustulose, thick and heavy spiral keels at periphery, and a third, much lighter cord on the base, partly covered by next whorl; strong, prosocline, axial riblets on sutural ramp, fading out abapically towards spiral keel; and irregular, short, elongated or rounded granules that cover the abapical part of the whorl and may extend in between the two peripheral keels; protoconch/teleoconch transition sharp, with a lamellar terminal varix and a deeply indented sinusigera notch (Fig. 3K, L). On early teleoconch whorls sculpture consisting of closely-set axial ribs intersected by three spiral cords together forming beaded intersections, abapicalmost cord stronger, forming an angular projection at intersection with ribs; axial and spiral sculpture becoming weaker on exposed parts of subadult and adult whorls, until an almost smooth last whorl; last whorl with weakly defined basal cord encircling convex base bearing six well defined, raised cords. Siphonal canal very short, broadly open, not constricted. Aperture circular-ovate, ~32% of shell height. Anal canal indistinct. Columella concave with very thinly callused columellar lip. Outer lip of aperture slightly thickened, but not forming a terminal varix, subvertical on periphery, regularly convex on base. Shell surface smooth and shiny, as if waxed, color overall rich honey to golden brown with broad, opaque, irregular white fascioles extending from suture to suture at irregular intervals; adapical and basal cords with alternating white and honey blotches at regular intervals; columellar side of anal and columellar callosity white, parietal callosity transparent. Tip of teleoconch (first whorl) white, protoconch dark brown. Dimensions: 7.97 × 3.40 mm. Average 6.83 ± 1.47 mm (n = 12).

**Operculum.** Subcircular, paucispiral, comprising three whorls. Nucleus large, subcentral, ~72% of operculum length.

**Figure 4.**
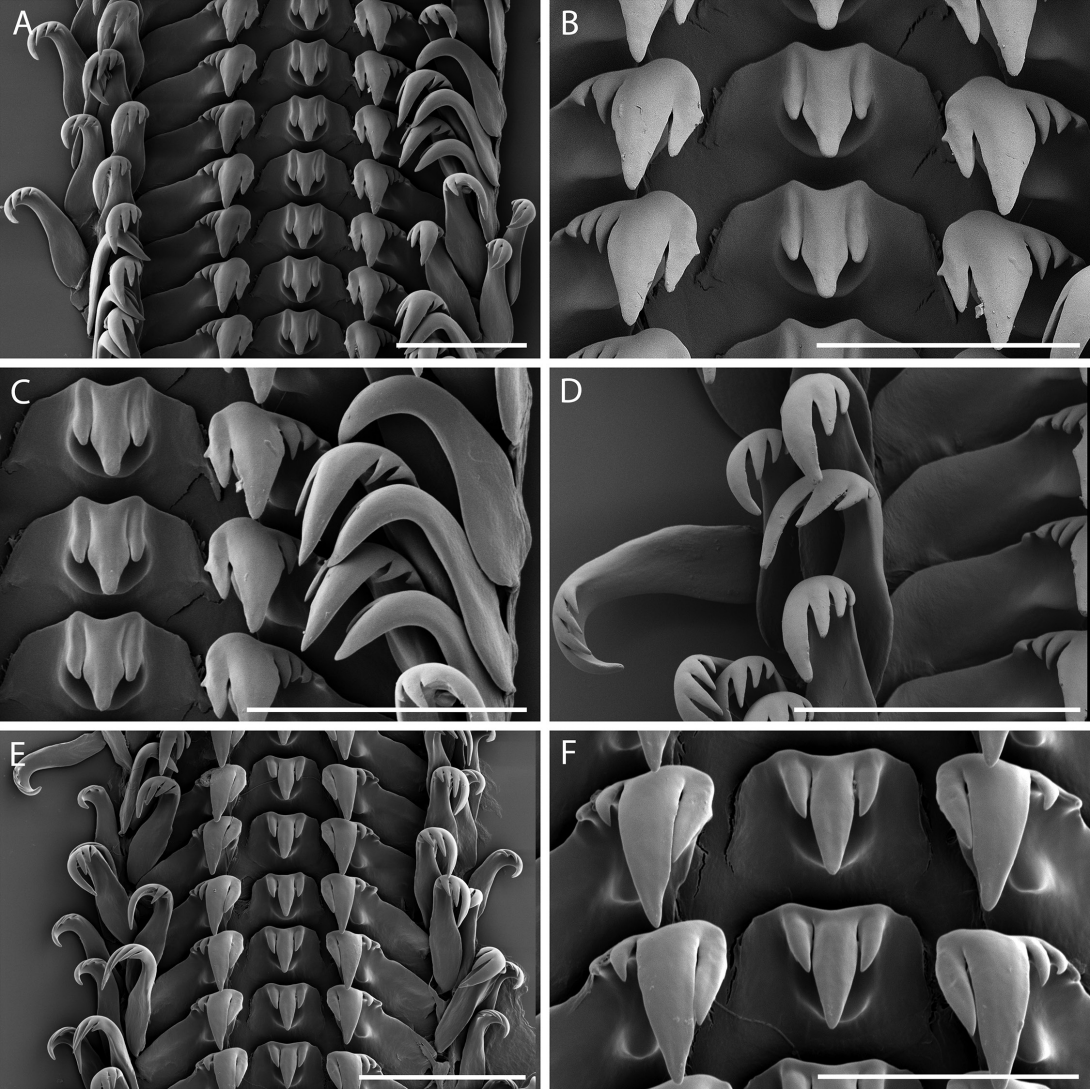
Radula morphology of *Limatium aureum* sp. n. **A–D** Holotype, MNHN IM-2013-42460, Austral Islands, Maria **E, F** Austral Islands, Pointe Kauira, RAPA 2002 stn. 36 **A** Radular ribbon **B** Detail of rachidian and lateral teeth **C** Detail of half row, showing outer edges of lateral and marginal teeth **D** Detail of marginal teeth, showing inner edges **E** Radular ribbon of second specimen, showing variation in length of cusps **F** Detail of rachidian and lateral teeth, showing variation in width of rachidian and in length of cusps. Scale bar: 50 µm (**A, C, D, E**); 40 µm (**B**); 30 µm (**F**).

**Radula.** Rachidian (Fig. 4A–C, E, F) with roughly hexagonal to septagonal basal plate, squarish to rectangular, broader than tall, with elevated central portion with rounded, U-shaped lower margin. Cutting edge bearing one strong central and two lateral long, dagger-like pointed cusps; central cusp up to twice as long as lateral cusps. Lateral teeth (Fig. 4A, B, C, E–F) with short lateral extensions, roughly 1.5 times length of cutting edge. Cutting edge with large central, pointed cusp and single, large pointed inner cusp and two to four tapering outer denticles. Thickened buttress extending down face of marginal tooth slightly outside central cusp, terminating in prominent, bluntly rounded peg roughly halfway down face (Fig. 4F). Marginal teeth (Fig. 4A, C–D, E) similar in shape and denticulation. Inner marginal teeth with large pointed central cusp, two to three tapering inner pointed cusps and one to two outer pointed denticles. Outer marginal teeth with large, pointed, central cusp, two to three tapering inner pointed cusps and a smooth, acuspate outer edge.

####### Distribution and ecology.

Austral Islands and Tuamotus (French Polynesia); Loyalty Islands, mainland New Caledonia, and Coral Sea. Known only from the material examined. Collected alive in 10–100 m, empty shells to 560–1150 m were undoubtedly carried downslope.

####### Remarks.

The holotype is “untypical” in the sense that it is an unusually broad specimen that, however, seems to be connected by morphologically intermediate specimens to forms that are more slender and with a strong cord delimiting the basal disc. All these specimens share a color pattern of alternating white and honey blotches on the subsutural and basal cords, in addition to ill-defined axial white fascioles, on an overall rich, golden honey background. Another type of variation comes from the extension/persistence of the spiral cords on subadult/adult whorls – with specimens almost completely smooth on the periphery of the last whorl and others with strong spiral cords persisting onto the last whorl.

An empty shell collected from Tonga (BORDAU 2 stn. DW1512, between Eua and Tongatapu, 21°19'S, 175°01'W, 183–184 m) (MNHN IM-2014-6922, Fig. 3D) is unusually tall and narrow, with spiral and axial sculpture persisting onto the last whorl; its color pattern, however, is very similar to that of *L. aureum* and we tentatively consider it to belong to that species.

###### Limatium

Taxon classificationAnimaliaSorbeoconchaCerithiidae

sp.

Fig. 3I, J, M

####### Material examined.

**PHILIPPINES**: MUSORSTOM 3 Stn. DR117, W of Mindoro, 12°31'N, 120°39'E, 92–97 m, 1 lv (MNHN IM-2014-6930, Fig. 3I). **VANUATU**: SANTO 2006 stn. DS103, W of Tutuba I., Vunatavoa Bay, 15°34.1'S, 167°16'E, 10–80 m, 2 dd (MNHN IM-2014-6931, IM-2014-6932, Fig. 3J, M).

####### Remarks.

Three specimens (Fig. 3I, J, M) show an overall resemblance to *L. aureum*, but differ in a manner that we think they are not conspecific. The three specimens from the Philippines and Vanuatu are subadults; their color pattern does not have the articulated white and golden honey spiral cords. The specimens from Vanuatu have a single cord on the base versus five in the specimen from the Philippines, and these two specimens may not even be conspecific. The protoconch of a Vanuatu specimen (Fig. 3M) is distinctly smaller than in *L. aureum*, consisting of only two whorls, with fewer and shorter axial riblets on the ramp, and simpler, less ornamented spiral keels. No specimen of this or any other *Limatium* has been obtained in the Philippines despite extensive sampling by lumun-lumun for the commercial shell trade (G. Poppe and S. Tagaro, pers. comm.).

### Phylogenetic analysis

Bayesian analysis of the concatenated COI and 16S dataset recovered the monophyly of the Cerithiinae and Bittiinae, although the latter is not statistically supported. *Limatium* is monophyletic (PP = 1) and is robustly supported (PP = 0.99) within the Bittiinae as the sister group to *Cacozeliana* Strand, 1928. *Ittibittium* and *Pictorium* are also monophyletic both with robust support (PP = 1). The clade including the type species of *Bittium* Gray, 1847 [*B. reticulatum*, *B. latreillei* (Payraudeau, 1826), *B. simplex* (Jeffreys, 1867)] also received high support (PP = 1).

## Discussion

Houbrick’s (1993) generic review of the Bittiinae published 25 years ago remains the authoritative resource for comparative anatomy and systematics of bittiines. At that time, Houbrick (1993) recognized nine genera in the *Bittium*-group: *Bittium*, *Argyropeza*, *Bittiolum*, *Cacozeliana*, *Ittibittium*, *Lirobittium*, *Neostylidium* [then as *Stylidium*], *Varicopeza*, and *Zebittium*; *Cassiella* was identified as a possible member but its placement uncertain given the paucity of anatomical data. In 2006, Bandel established a separate subfamily for *Argyropeza*. Since then, the Bittiinae has been expanded (MolluscaBase 2018) to include the Recent genera *Cerithidium* (provisionally excluded by Houbrick 1993), and *Pictorium*.

Despite the absence of a diagnostic feature that allows unambiguous placement in the subfamily (Strong and Bouchet 2013), the common perception of bittiines is that they are small in adult size, turreted, with a predominating beaded or spiral sculpture, and cream, gray, tan to dull brown in color. This concept of bittiine teleoconch morphology was expanded by the description of the genus *Pictorium*, based on a small type species formerly placed in *Cerithium*, with a pupoid, brilliantly colored reddish-purple shell (Strong and Bouchet 2013). *Limatium* species differ from all other bittiines in their smooth, glossy, polished shells with a rich honey to golden brown background color. This unique shell morphology is unknown in the Cerithiidae and further expands the conchological concept of the subfamily. The distinctive white fascioles also may be a diagnostic feature of the genus, but further comparative material is required to be certain, particularly of the suspected additional species from Vanuatu and the Philippines. Clearly, more material, and more sequencable material, is needed to understand intraspecific variation within *Limatium*.

In addition to shell morphology, *Limatium* differs from known bittiines in several unique features of the radula. Most possess a rachidian with an attached basal portion and a freely projecting face. The face of the tooth has a central to basal constriction that can be quite strongly developed in some species. Thickenings at the lower, outer corners of the tooth extend onto the radular membrane beyond the sides of the tooth. The upper projecting margin of the tooth often forms a prominent crest from below which the teeth project. The cutting edge spans the entire anterior edge of the tooth, or the majority of it, and bears a single, strong central cusp and two to three denticles on each side (e.g., Houbrick 1980b, 1993, Gofas 1987, Ponder 1993, Hasegawa 1998). The rachidian of *Limatium* has a basal plate that is hexagonal to septagonal in shape, with a central elevated portion that has a U-shaped, rounded lower edge roughly midway down the face of the tooth. The cutting edge is restricted to this elevated portion, and bears only three dagger-like cusps. This configuration is unknown among the Bittiinae, and even more broadly among the Cerithiidae. In terms of operculum morphology, most bittiines are characterized by a multispiral operculum (Houbrick 1980b, 1993, Gofas 1987, Hasegawa 1998); *Bittiolum* and *Cassiella* differ in possessing an ovate, paucispiral operculum with a small nucleus (Houbrick 1993, Ponder 1993). In contrast, *Limatium* possesses a subcircular, paucispiral operculum of only ~3 whorls and a large nucleus. Argyropezinae conform to the bittiine configuration of rachidian morphology and possess a circular, multispiral operculum (Houbrick 1980a).

The phylogenetic analysis supported *Limatium* and *Cacozeliana* as sister taxa, although more comprehensive taxon sampling is required to assess the affinities of bittiine genera. The analysis confirmed that *Bittium*, as presently conceived is polyphyletic (Strong and Bouchet 2013). Inclusion of the type species, *Bittium reticulatum*, for the first time allows us to tie the genus-group name to a clade including *B. latreillei* and *B. simplex*. As in the analysis of Strong and Bouchet (2013), *Bittium impendens*, only cautiously retained in *Bittium* by Houbrick (1993), is robustly supported as the sister group to *Pictorium*; *Bittium glareosum* is the sister to them but its placement is not supported. Additional sampling is required to resolve the affinities of Indo-Pacific species currently placed in *Bittium*, with *Bittium* *s.s.* possibly retained only for species from the Atlantic.

*Limatium* is exceptionally rare, represented in museum and personal collections by a scant 108 specimens known to us as enumerated herein, only 16 of them collected alive. As described in Strong and Bouchet (2013), *Pictorium* also had been rare in museum collections and had never been collected alive prior to the 1980's. Like *Limatium*, the latter genus is also small and found in steep, hard-bottom habitats that are too deep for diving and too steep for dredging. The use of lumun-lumun and tangle nets in the commercial shell trade in the Philippines, and the adoption of new collecting techniques as brushing baskets and suction sampling in biodiversity surveys (Albano et al. 2011), have revolutionized access to these challenging habitats. While the number of specimens of *Pictorium* rose dramatically particularly since 2004, the number of *Limatium* specimens also has increased but not so dramatically, and live collected specimens remain elusive. The unique radular morphology of *Limatium* suggests a life habit different from that of most other bittiines, which may explain why they remain so tantalizingly rare.

**Figure 5.**
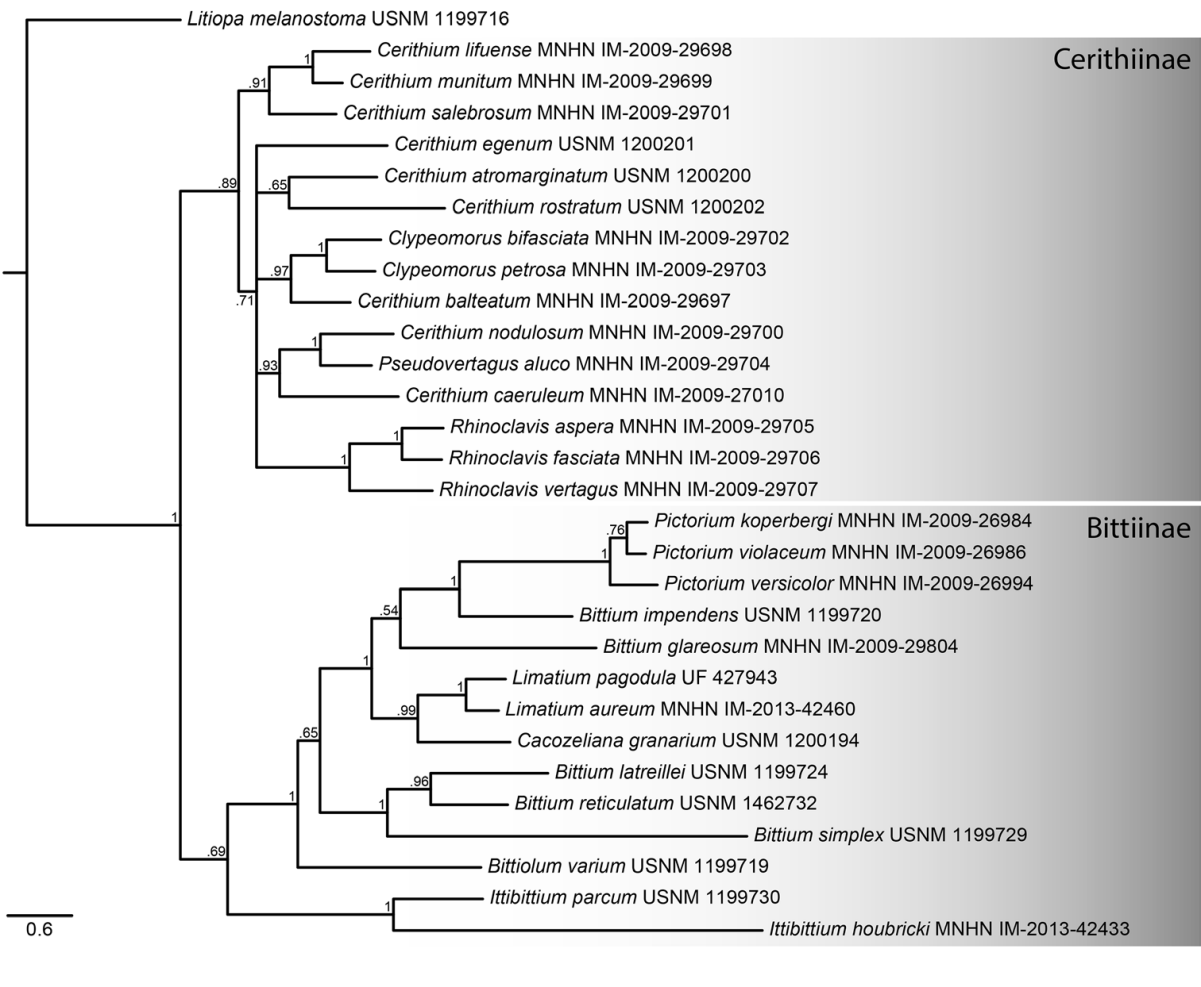
Phylogeny of Cerithiidae based on Bayesian analysis of partial COI and 16S sequences. Catalogue numbers for vouchers indicated after species name. Posterior probabilities greater than .50 are shown at the nodes. See Table 1 for details.

## Supplementary Material

XML Treatment for Limatium

XML Treatment for Limatium pagodula

XML Treatment for Limatium aureum

XML Treatment for Limatium
